# Supporting parents of children with learning disorders: a systematic review of intervention strategies

**DOI:** 10.3389/fpsyg.2025.1536894

**Published:** 2025-05-30

**Authors:** Giulia Carlotta Guerra, Maria Teresa Positano, Alessandra Sperati, Ilenia Passaquindici, Maria Grazia Logrieco, Francesca Lionetti, Maria Spinelli, Mirco Fasolo

**Affiliations:** ^1^Department of Neuroscience, Imaging and Clinical Sciences, University of Studies “G. d’Annunzio” Chieti-Pescara, Chieti, Italy; ^2^Department of Human Sciences, University of Foggia, Foggia, Italy; ^3^Department of Nervous System and behavior Sciences, University of Pavia, Pavia, Italy; ^4^Department of Psychology, University of Studies “G. d’Annunzio” Chieti-Pescara, Chieti, Italy

**Keywords:** learning disability, intervention, parent education, support group, children, parent training

## Abstract

**Introduction:**

Specific Learning Disorder (SLD) is a neurodevelopmental condition affecting 5–15% of children worldwide, typically involving difficulties in reading, writing, and/or mathematics. Dyslexia is the most common subtype. These disorders impact both children and their parents, highlighting the need for emotional and educational support to help families manage SLD-related challenges. Although various parenting programmes exist, little is known about which features make some interventions more effective than others.

**Methods:**

This systematic review assessed the effectiveness of psychotherapeutic and psychoeducational parenting interventions. A comprehensive search was conducted across multiple databases for studies published between 1950 and 2024, following PRISMA guidelines. From 1,519 records, 10 studies met the predefined inclusion criteria.

**Results:**

The included studies showed that parental support interventions improved parents’ emotional well-being and coping strategies. These improvements were linked to better academic, behavioural, and social outcomes in children. Interventions differed in format and delivery, but all focused on strengthening parental skills and knowledge.

**Discussion:**

Parenting interventions that combine emotional and educational support appear beneficial for both parents and children. They foster environments that support children’s development. Further research is needed to identify the most effective components and delivery methods across diverse populations.

## 1 Introduction

Specific Learning Disorder (SLD) is a neurodevelopmental condition characterised by underachievement in school, typically emerging in the early years of primary school. It is two to three times more common in boys than girls (American Psychiatric Association, D, 2013; Ghaffari et al., 2022; Grigorenko et al., 2020). While children with SLD have normal levels of intelligence (i.e., IQ > 70), they face challenges in various areas such as reading, mathematics, and written expression (Lyon et al., 2003; Tan et al., 2024). Additionally, these children may experience difficulties with attention, motor skills, and social functioning (Lerner, 2003; Tan et al., 2024). Although some children with SLD may struggle in only one academic area, deficits in reading, mathematics, and writing often co-occur, with reading difficulties being the most common (i.e., dislexia; Willcutt et al., 2019; Shaywitz, 1998; Tan et al., 2024). These children are also at higher risk for emotional and behavioural issues, including anxiety, depression, low self-esteem, compulsive behaviours, hostility, and emotional instability. In some cases, there may be increased risks of suicide and criminal behaviour (Faust, 2003; Hu et al., 2020; Xiao et al., 2023; Tan et al., 2024). These difficulties affect not only the child but also their parents, who must manage the academic, emotional, and behavioural needs of their children. Parents of children with SLD often experience significant stress as they oversee their child’s learning activities and try to address the various needs they face (Finardi et al., 2022). Common challenges include tension around homework (Waggoner and Wilgosh, 1990), feelings of guilt, and emotional detachment (Dyson, 2010; Latson, 1987). Other frequent struggles involve exhaustion, sadness (Waggoner and Wilgosh, 1990), difficulty accepting the learning disability (Dyson, 2010), and ongoing concerns about their child’s future (Hanneil, 1995; Brock and Shute, 2013). Moreover, because children with SLD often face poor long-term social and vocational prospects (Aro et al., 2019; Grigorenko et al., 2020) and low self-esteem (Brock and Shute, 2013), parents may also feel additional stress regarding their child’s social connections and future career opportunities (Tan et al., 2024). These concerns are further compounded by the child’s struggles with their learning difficulties (Hanneil, 1995), as well as behavioural issues such as distractibility, hyperactivity, poor attention span, poor listening skills, and difficulty completing tasks (Fuller and Rankin, 1994; Latson, 1987). Finally, other challenges include adapting to the mainstream school environment (Tan et al., 2024). External stressors, beyond the family’s control, also contribute to parental strain. These include interactions with teachers, other professionals, and the school system (Dyson, 2010; Hanneil, 1995; Waggoner and Wilgosh, 1990). These parents have also to deal with delayed and costly assessment procedures, and with the high cost or limited availability of tutoring programmes (Dyson, 2010), and lack of social support (Dyson, 2010; Waggoner and Wilgosh, 1990). All these factors could impair the quality of parent–child interactions and increase family stress, with consequent conflict and straining marital relationships (Bonifacci et al., 2014; Heiman, 2021). As a result, even if families of children with learning disabilities are not inherently less functional than families of children without disabilities (Brock and Shute, 2013; Dyson, 2010), it is well recognised that they could experience many stressors that, combined with those related to the child’s learning difficulties, could affect parents’ emotional well-being and place them at higher risk of developing mental health problems (Bonifacci et al., 2014; Carroll, 2013; Karande and Kulkarni, 2009). Therefore, providing support to parents of children with SLD is crucial to help them cope with the stressors and daily academic and cognitive challenges they face (Fernandez-Alc’ Antara et al., 2017). This support can come in the form of clinical or educational interventions aimed at enhancing parental coping skills and improving the overall family dynamic. Consequently, several support programmes specifically targeting parents of children with SLD have recently been implemented. Effective parental support through targeted interventions has been shown to strength protective factors, such as parenting skills, manageable stress levels, and better management of school environment, all of which are key to ensuring a stable foundation for the child’s well-being and future adjustment (Brock and Shute, 2013). These interventions vary in nature, such as online or in-person formats, and focus on different themes considered relevant. Emotion-focused programmes aim to reduce parental stress and strengthen the parent–child relationship (Shechtman and Gilat, 2005; Tan et al., 2024), while training and coaching interventions provide parents with practical strategies to better support their children (Ghaffari et al., 2022; Ruan et al., 2022). Behaviour management techniques help parents develop coping skills and manage child learning challenges (Brock and Shute, 2013; Ryback and Staats, 1970), while structured programmes, offered both online and in person, address emotional and behavioural difficulties (George et al., 2011; Multhauf et al., 2016). Additionally, some studies emphasise the importance of social and financial support in alleviating parental stress, highlighting the multifaceted nature of effective interventions. Although the literature on this topic has grown in recent years, it remains unclear which characteristics make some interventions more effective than others in improving parents’ well-being and skills. To address this gap, the present systematic review aims to identify the interventions reported in the literature and highlight their specific characteristics in relation to the reported outcomes.

## 2 Research methods

This systematic review was conducted following the Preferred Reporting Items for Systematic Reviews (PRISMA) guidelines, including 10 research articles published between 1950 and 2024.

### 2.1 Eligibility criteria

After identifying the keywords, inclusion and exclusion criteria were established to clarify the search more notable and useful nature in relation to the subject to be dealt with. The researchers set out the following inclusion criteria: (I) The intervention is targeted at primary caregivers of children diagnosed with Specific Learning Disorders. (II) Children must be between 4 and 17 years old. (III) The intervention can have any type of approach (e.g., cognitive behavioural, systemic, dynamic), (IV) single cases are included. (V) On the intervention aimed at parents, the child must not be present. (VI) The articles must be in full-text journal and published between 1950 and 2024, because the literature on SLD at an international level starts from 1950. (VII) The article can be written in English or Italian language. The researchers have established the following exclusion criteria: (I) Children who are diagnosed or co-morbid with ADHD, Autism, Disability. (II) Parents with psychiatric diagnoses. (III) Articles: Reviews, Meta-analyses, books, conferences. (IV) Interventions that include teachers or other family members in addition to primary caregivers. (V) Interventions aimed at parents that includes the child. Then, studies were grouped according to specific variables such as intervention (online, in-person, group, individual), focus (information, emotions) and outcome (positive or negative effectiveness).

### 2.2 Information sources and search strategy

Review articles published between 1950 and 2024 were searched online via electronic databases such as Web of Science, Scopus and PubMed. The last search was conducted on 11 October 2024. First, guided by our research aim and the insights emerged from the reviewed literature, we defined the search terms to identify articles relevant to the topic, according to the PICO criteria (Population, Intervention, Comparison, and Outcome). As the population, we set “parents of children with DSA”; as an intervention, we set the use parent training and support; for the comparison, we used words that refer to other neurodevelopmental disorders; and finally, as an outcome, we set improvement of the well-being and empowerment of parents of children with DSA. The papers were searched by using the keywords, namely “specific learning disorder” OR “specific learning impairment” OR dyslex* OR dyscalcul* OR dysgraph* OR dysorthograph* AND parent* OR mother* OR father* OR famil* OR caregiver*AND support* OR intervention* OR training* OR counsel* OR empower* OR “parental involvement” OR “family support” NOT autism OR ADHD OR “intellectual disability.” These key keywords are further diversified so that article search can be done more widely. Key keywords and relevant variations have been identified and applied, including synonymous or terms with similar meanings. These were then combined with Boolean operators (AND, OR) to optimise the search strategy (see Supplementary Table S1).

### 2.3 Selection process and data collection process

**Figure 1:**
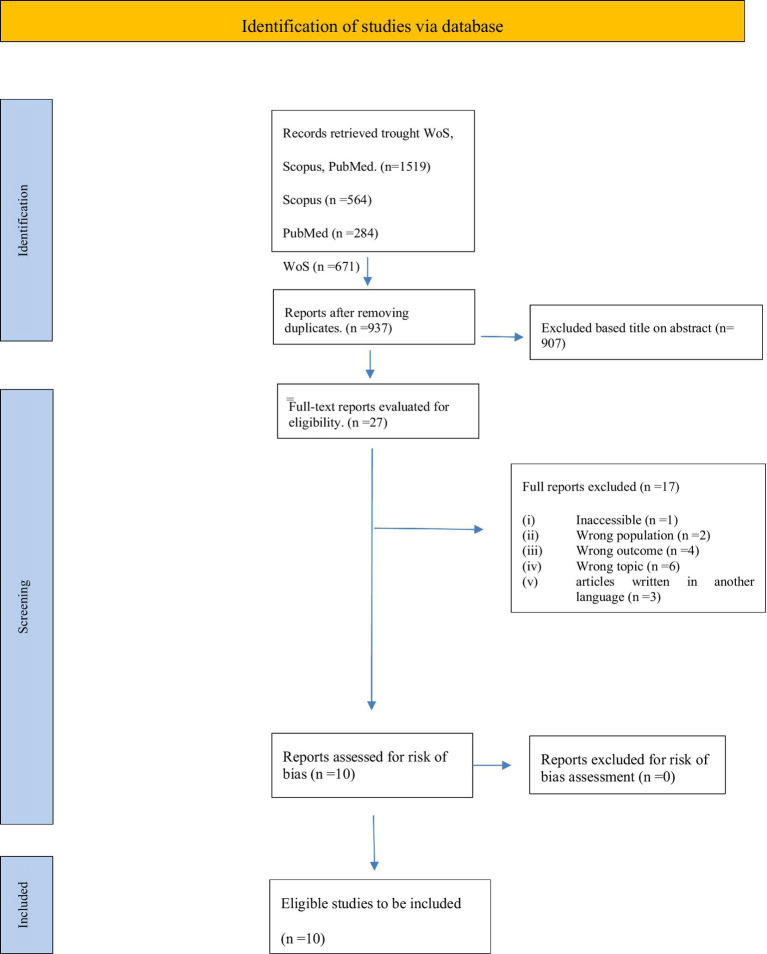
PRISMA 2020 flow diagram for new systematic reviews which included searches of databases, registers, and other sources.

The search identified a total of 937 potentially eligible studies, excluding duplicate studies, of which 10 met the inclusion criteria. The search engines produced different numbers of articles: 665 in Web of Science, 280 in PubMed, 557 in Scopus. Article selection was carried out with Zotero, which allows two authors to work synchronously to review the articles produced by searching the three search engines. The first stage of review was done by reading the title and abstract of each article and excluding those that did not meet the inclusion and exclusion criteria. After this first stage, 27 articles remained. The two authors then worked individually, dividing the articles and systematically reading them one at a time. Sixteen articles were excluded because they did not meet the inclusion and exclusion criteria. One article was excluded because it could not be found. The remaining 10 selected articles were systematically reviewed and data on the general characteristics of the studies were extracted. These characteristics included: study, methodology, types of intervention, outcomes, limitations and recommendations. Data and information were extracted from each study independently by the two authors (Table 1). Finally, the risk of bias was analysed using the Cochrane Risk of Bias Tools 2 (ROB2) method (see Supplementary Table S2). In cases of disagreement, a consensus approach was adopted that included a third author. For each study, the year of publication and references were extracted. The following information were coded: (i) description of the Sample Parents and Sample Children (age, gender, country, and diagnosis), (ii) description of the interventions (method—teaching approach or emotional and relational), control group, duration, frequency, and (setting—group, individual, online or in-person), and (iii) outcomes (finding) and (iv) limits. The entire process of article selection is shown in Figure 1.

**Table 1:** Summary of articles included in the current literature review.

Author	Sample parents	Sample children	Country	Diagnosis	Control group	Type of intervention	Duration	Findings	Limits
Abd Rauf et al. (2020)	50 parents (94% female)	50 children	Malaysian	Dyslexia	No	Grouped into peer-to-peer, Government support, Social support, Financial support	The duration of the intervention is not specified	No differences found: The type of support does not differentially impact parents of children with dyslexia. Equal benefits: All types of support provide benefits to parents.	The study does not compare the type of support received based on geographical location or family/personal circumstances. The sample size of the study is limited to only 50 participants.
Brock and Shute (2013)	57 mothers	57 children (6–12 years old)	Australia	learning disabilities	Yes	Online skill-based training programme focused on: education about dyslexia and other Learning disabilities Coping strategies for parents Child management strategies.	Four 2-h sessions held weekly.	The intervention reduced mothers’ stress levels.	Parental stress reduction was statistically significant but modest; nearly half of parents still reported clinical stress levels after the program.
George et al. (2011)	5 parents	5 children (school age children)	United Kingdom	learning disabilities	No	Group intervention (presence). Incredible Years Parent Training Programme adapted for use with parents of school-aged children with moderate to severe learning disabilities.	The intervention took place in 12 weekly sessions of 2 h each.	Reduced parental stress. Slight increase in children’s behavioural problems. Fewer inappropriate behaviours, more positive behaviours observed. Parents mostly “satisfied” with the programme.	The study is a small pilot with a high dropout rate (44%) and no control group, limiting generalisability and isolating intervention effects.
Ghaffari et al. (2022)	54 mothers (25–50 years old)	children (7–12 years old)	Iran	SLD	Yes	Occupational Performance Coaching (OPC) model for all mothers and four additional 4QM (Four Quadrant Model of Facilitated Learning) training sessions for mothers in the experimental group.	OPC interventions consist of ten 60-min sessions provided once a week. The OPC + 4QM interventions consist of fourteen 60-min sessions (ten sessions for OPC + four sessions for 4QM) provided once a week.	**-**OPC intervention: Improves participation in out-of-school activities for children with SLD. **-**4QM addition: Enhances mothers’ ability to meet children’s learning needs.	The study included only mothers.
Multhauf et al. (2016)	39 parents (M age = 43.2)	39 children (M age = 8.8)	Germany	SLD	Yes	CBT-intervention on focus group (presence)	5 parent training sessions. The total duration of the intervention was 3 months	Reduction in parental stress: Significant decrease in stress levels. Improved coping: Mothers better managed children’s literacy difficulties. Enhanced parent–child interactions: Reduced conflict and improved learning situation management. Sustained effects: Benefits maintained three months post-programme.	The intervention significantly reduced parental stress and improved mothers’ ability to manage children’s literacy difficulties. It also enhanced parent–child interactions, reducing conflict and improving learning management. These positive effects persisted three months after the programme ended.
Ryback and Staats (1970)	4 parents with ages ranging from 31 to 41 years	4 children involved had an average age of 10 years and 7 months	United States	Reading deficits	NO	The state’s Motivation-Activating Reading Technique (SMART), an approach that uses parents as behavioural technicians to help their children overcome reading difficulties.	From 35 to 65 h of training for the children with reading deficits	The results showed significant improvement in children’s reading skills, demonstrating that parents, with training and supervision, can effectively support reading interventions. This highlights the crucial role of parents in addressing reading difficulties, even severe ones.	Small sample size. Variability in children’s starting conditions limits conclusions about the intervention’s effectiveness for all children with reading difficulties. No control group was mentioned.
Ruan et al. (2022)	63 mothers, 8 fathers	110 children (primary and secondary school)	China	dyslexia	Yes	Massive open online course (MOOC) booklet. Training group. The booklet included five units.	1 month	Parent coaching improved reading and spelling skills in both dyslexic and non-dyslexic children, emphasising the role of parental support in literacy development.	Parental completion was monitored via self-report only, with no other measures used. Parents learned the material independently. The quality of parental teaching was not well monitored, potentially affecting coaching effectiveness. The study had a small sample size.
Shechtman and Gilat (2005)	95 mothers: 49 in the counselling group (M = 38.67) and 46 in the educational group(M = 36.33) and an equal number of fathers who completed the questionnaires for counselling group (M = 40.67) and for educational group (M = 39.76)	95 Children ranged in age (M = 8.62).	Israel	Learning disabilities (LD)	No	Group intervention (presence). The counselling intervention focused mainly on emotional support and sharing experiences between parents, rather than simply providing information about their children’s learning problems. The psycho-educational intervention was structured to provide information and training to parents.	The intervention consisted of 90 min sessions	Parents in the psycho-educational intervention group showed no significant improvement in stress, coping, or relationships with their children. In contrast, parents in the counselling group experienced significant positive changes, particularly in their ratings of children with LD.	Further research is needed on family dynamics and support for parents of children with learning disabilities. Psycho-educational interventions with counselling should be tested, including long-term follow-up. Fathers should be involved, and family interactions explored for their impact on well-being.
Tan et al. (2024)	69 parents (97% mothers) (M = 40.72)	69 Children (M = 9.97)	China	LD	Yes	A group mindful parenting (MP) programme. The approach focused on experiential inquiry-based learning, including formal mindfulness practices (body scan, mindful breathing, meditation, etc.) and group sharing. The programme was tailored to address the specific challenges of parenting children with learning disabilities (LD). Additional psycho-educational content was included, with a focus on emotional regulation and self-care for parents.	The duration of the mindfulness intervention described in the study is 8 weeks. Two hours per session, held once a week.	The MP group experienced reduced parenting stress, enhanced emotional awareness, better attention to children, and increased self-compassion.	The intervention lasted 8 weeks, which may not be long enough to assess the long-term effects of mindfulness on parenting stress. Variability in participants’ experiences and responses may have influenced the results, and it was not specified how this was controlled for.
Uslu et al. (2006)	81 parents in 3 groups.	42 children	Turkey	specific learning disorders LD	Yes	The group intervention with parents in the paper was a psychoeducational programme with information sessions on how learning difficulties affect children’s behaviour and learning.	The programme took place over 1 month, with weekly sessions.	The results showed a significant improvement in children’s reading skills, indicating that parents can be effective behavioural technicians with proper training and supervision. This study highlights the key role parents can play in treating reading difficulties, even in severe cases.	Short treatment duration, no parental psychopathology assessment, and unconsidered ADHD comorbidity may have affected results. Longitudinal studies needed to assess long-term psychoeducation effects. Recommend multidisciplinary support for families.

### 2.4 Reporting bias assessment

The results show several trends in the risk of bias in different studies, influenced by randomisation methods, blinding and management of missing data:

1. Low risk of bias:

Ghaffari et al. (2022), Multhauf et al. (2016), and Tan et al. (2024), George et al. (2011) and Uslu et al. (2006) showed a low risk of bias due to the use of randomisation and strict management of missing data. Although Multhauf et al. (2016) and Tan et al. (2024) did not use full blinding, which limits interpretation in the area of power and detection, their methods were considered robust.

2. Unclear risk of bias:

Ryback and Staats (1970), Shechtman and Gilat (2005), Ruan et al. (2024) showed an uncertain risk of bias due to shortcomings in randomisation and blinding. These aspects could affect the validity of the results and introduce elements of uncertainty.

3. High risk of bias:

No article had a high risk of bias.

The entire process of article selection is shown in Figure 1.

## 3 Results

### 3.1 Study selection

The initial search identified a total of 1,519 articles. After excluding duplicate studies, screening for relevant topics and reading abstracts and full texts, 27 articles were found to meet the study criteria. Of these, 10 studies were included in the qualitative analysis. The study selection process is presented in the PRISMA diagram (Figure 1), which illustrates each stage of the screening process, including the reasons for excluding articles at each stage.

### 3.2 Sample characteristics

The qualitative analisys included 10 studies conducted between 1950 and 2024, with samples that varied in age, nationality and participant characteristics. All the studies involved parents of children with specific learning difficulties (SLD), including dyslexia, dysgraphia, dysorthographia and dyscalculia. The children in the trials were aged between 6 and 12 years. 5 out of 10 studies targeted mothers only (Brock and Shute, 2013; Ghaffari et al., 2022; Ruan et al., 2022; Shechtman and Gilat, 2005; Tan et al., 2024), the other 5 targeted both parents (Abd Rauf et al., 2020; George et al., 2011; Multhauf et al., 2016; Ryback and Staats, 1970; Uslu et al., 2006). The countries in which the studies were carried out are: Asia, particularly Malaysia (Abd Rauf et al., 2020), Iran (Ghaffari et al., 2022), China (Ruan et al., 2022; Tan et al., 2024), Turkey (Uslu et al., 2006), and Israel (Shechtman and Gilat, 2005); Europe, particularly the United Kingdom (George et al., 2011), and Germany (Multhauf et al., 2016); Oceania, particularly Australia (Brock and Shute, 2013); and the Americas, particularly the United States (Ryback and Staats, 1970).

### 3.3 Selection and topic

#### 3.3.1 Online interventions

Four studies included in the review used online interventions to support parents of children with learning disabilities (Abd Rauf et al., 2020; Brock and Shute, 2013; Ruan et al., 2022; Tan et al., 2024). Abd Rauf et al. (2020) intervention aimed to investigate different types of support for parents of children with dyslexia. The intervention involved online data collection through the administration of several questionnaires, such as Dyslexia. My Parents Support Group; Rakan Disleksia (RD); Pertubuhan Sokongan Ibubapa Dyslexia Malaysia. Data collection took 2 months. The study identified four main types of support needed: social and family support (advice and encouragement from family, friends or the community; reduces stress and depression, improves lifestyle and promotes parent–child interaction), peer-to-peer support (parents with similar experiences come together to share knowledge, experiences and mutual support; this support helps to reduce isolation and increases parental confidence), government support (including availability of specialists, training programmes for parents and teachers and dyslexia awareness campaigns), financial support (essential to access additional educational services and meet therapy costs). The intervention used by Brock and Shute (2013) consisted of four weekly online interactive sessions, each lasting 2 h. The sessions had three aims: to inform parents about dyslexia and other learning difficulties, to provide them with techniques for managing stress and coping with the emotional challenges of parenting, and to teach strategies that would enable them to support their children with learning difficulties, both at home and at school. Ruan et al. (2022), on the other hand, described a one-month online intervention delivered through a booklet, i.e., the Massive Open Online Course (MOOC). The MOOC is divided into 5 units (e.g., basic concepts and knowledge of learning disabilities, practical strategies to support children’s literacy, techniques to improve parental involvement in education) and aims to provide training on learning disabilities for parents, with learning materials to be completed independently. Finally, Tan et al.’ (2024) implemented a Mindful Parenting (MP) programme based on experiential learning and awareness, to address the challenges faced by parents of children with learning disabilities. The intervention included both mindfulness practices (e.g., body scan, mindful breathing, and meditation) and psycho-educational components (e.g., focus on emotional regulation and self-care for parents). It consisted of 8 weekly sessions of 2 h each. The results of all three studies show the effectiveness of online interventions for parents. In particular, both Brock and Shute (2013) and Tan et al. (2024) interventions significantly reduced parenting stress. In addition, Tan et al. (2024) also found an improvement in parents’ awareness and attention to their children’s emotional and behavioural needs, an improvement in parents’ ability to treat each other with kindness and understanding, reducing feelings of guilt or frustration, and an improvement in the parent–child relationship. Brock and Shute (2013) also found an effect on educational attainment by taking action to manage children’s educational difficulties. Ruan et al. (2022) intervention had an effect on the educational domain by providing parents with reading and spelling strategies to support their children’s academic skills. Finally, the study by Abd Rauf et al. (2020) showed that all the types of interventions examined are needed to support parents of children with SLD.

#### 3.3.2 In-person interventions

Six studies included in the review used in-person interventions to support parents of children with learning difficulties (George et al., 2011; Ghaffari et al., 2022; Multhauf et al., 2016; Ryback and Staats, 1970; Shechtman and Gilat, 2005; Uslu et al., 2006). In their study, George et al. (2011) described an intervention based on the Incredible Years Parent Training for parents of children with moderate to severe learning difficulties. The intervention consisted of 12 weekly sessions of 2 h each. The sessions included group activities that included: group discussions to share experiences and challenges, practical exercises and role-playing to deal with common scenarios, and strategies to manage children’s challenging behaviours. The aims of the intervention were: to reduce parental stress, to promote appropriate behaviour in children, to improve parent–child interactions. Ghaffari et al. (2022) intervention involved the use of the Occupational Performance Coaching (OPC) model with or without the addition of the Four Quadrant Model of Facilitated Learning (4QM). Participants were divided into two groups (experimental: OPC combined with 4QM, control: OPC alone). The aim of the intervention was to evaluate the effectiveness of OPC with and without 4QM in improving children’s participation in extracurricular activities and mothers’ skills. Specifically, Occupational Performance Coaching (OPC) consisted of 10 sessions of 60 min each, followed by a one-month follow-up. The content of the sessions was: emotional support (caring, empathy, encouragement of the mothers); information exchange (performance analysis, learning strategies and available resources); structured process (definition of specific goals, planning and implementation of actions, review of progress). In contrast, the Four Quadrant Model (4QM; added in the experimental group) included 4 additional sessions during OPC (14 sessions in total for the experimental group). The 4 sessions included: direct maternal strategies (e.g., explicit instructions, demonstrations), indirect support for the child’s autonomous decision making, child-led strategies (e.g., self-instruction, mnemonics), child autonomy (e.g., problem solving, self-monitoring). Multhauf et al. (2016) described a cognitive behavioural therapy (CBT)-based intervention for parents, focusing on stress management and parent–child interactions. The intervention consisted of five sessions over 3 months. Each session focused on a specific aspect of managing children’s behaviours and difficulties. In particular, the sessions covered: managing parental stress (techniques for identifying and managing stress related to parenting, exercises to develop coping strategies), managing learning difficulties (practical approaches to supporting children with schoolwork and reading, techniques for dealing with frustration related to learning difficulties), improving parent–child interactions (strategies for reducing conflict; exercises to promote a positive learning environment; focus on how to manage stressful or tense situations related to school). Ryback and Staats (1970) intervention used the SMART (Motivation-Activating Reading Technique) technique, which involves 35–60 h of parent training (varying according to the individual needs of the children). The training was designed to train parents as ‘behaviour technicians’ to help children with reading difficulties. The intervention included: parent training (training in motivational and positive reinforcement techniques to encourage learning to read, expert supervision to ensure that parents applied the techniques correctly) and practical application (parents worked directly with their children to improve reading skills through specific exercises). Shechtman and Gilat (2005) described an intervention based on two approaches: the first was counselling, aimed at providing emotional support and sharing experiences. The second was psychoeducation, which aims to provide parents with practical information and educational support. The intervention consisted of group sessions of 90 min each. However, the total number of sessions is not specified. Finally, Uslu et al. (2006) described an intervention based on a “psycho-educational group program for parents,” which aims to address the effects of learning difficulties both at the behavioural level and on children’s academic performance. The intervention lasted 1 month with weekly sessions. The results of the in-person interventions described showed effectiveness in supporting parents of children with SLD. Ghaffari et al. (2022) study found an improvement in children’s participation in extra-curricular activities and an increase in mothers’ ability to respond to their children’s educational needs. George et al. (2011), Multhauf et al. (2016) and Shechtman and Gilat (2005) found a reduction in parental stress, improvements in parenting skills to manage children’s school difficulties, increased positive parent–child interactions and reduced conflict. In addition, George et al. (2011) also found a reduction in problem behaviour in children who appeared calmer. Finally, Ryback and Staats (1970) and Uslu et al. (2006) found significant gains in children’s reading skills and demonstrated that parents, with appropriate training, can be effective in supporting learning.

#### 3.3.3 Group intervention

The use of group interventions to support parents of children with SLD has proven to be effective. Abd Rauf et al. (2020) evaluated the effect of a group intervention consisting of different support modalities (peer-to-peer, governmental, social and financial) on parents of children with dyslexia. The results showed no significant differences between the different types of support but showed that all modalities provided comparable benefits to parents, suggesting an overall value of group support. Brock and Shute (2013) showed a reduction in parental stress levels, although almost half of the mothers continued to report clinical stress levels at the end of the intervention, suggesting only a partial benefit. Studies by George et al. (2011) and Uslu et al. (2006) evaluated psycho-educational programmes for parents of children with specific learning disabilities. The results showed a reduction in parental stress and an increase in positive child behaviour, with significant improvements in children’s reading skills. The group intervention by Ghaffari et al. (2022) showed effectiveness in improving children’s participation in extra-curricular activities and increasing mothers’ ability to respond to their children’s educational needs. A cognitive-behavioural group approach was used in the study by Multhauf et al. (2016), which led to a significant reduction in parental stress, an improvement in parent–child interactions, and an increase in the management of learning difficulties. Shechtman and Gilat (2005) compared two group interventions: one based on emotional counselling and one based on psychoeducation. Parents in the counselling group reported significant improvements in coping and relationships with their children, whereas the psycho-educational intervention showed no significant benefits. Finally, Tan et al. (2024) described a Mindful Parenting (MP) group programme that combined mindfulness practices with psychoeducational content. The programme led to a reduction in parenting stress, an increase in emotional awareness and greater self-compassion. Group interventions for parents of children with SLD demonstrate a valuable and multidimensional impact, highlighting their utility as a supportive resource for this population. Across various studies, group-based approaches appear to contribute significantly to parental well-being and child outcomes. In conclusion, group interventions represent a potent, adaptable, and effective framework for supporting parents of children with SLD. Their benefits encompass psychological, emotional, and practical dimensions, making them an invaluable addition to the repertoire of support strategies in this field.

#### 3.3.4 Individual intervention

The only study based on an individual intervention is that of Ryback and Staats (1970). The study involved four parents of children with reading difficulties and used a technique called Motivation-Activating Reading Technique (SMART). The intervention, which focused on each child and parent, differed from the others in that it focused on individualising the support. The results of the study showed a significant improvement in the children’s reading skills. The intervention emphasised the potential of the individual approach in achieving specific and targeted results, as well as the importance of active parental involvement in educational rehabilitation.

#### 3.3.5 Teaching approach interventions

Numerous interventions have attempted to provide practical tools and strategies to help parents support their children’s literacy and homework skills. Ryback and Staats (1970) introduced the Motivation-Activating Reading Technique (SMART) model, which uses parents as behaviour technicians to improve the reading skills of children with specific difficulties. The program, which involved between 35 and 65 h of training, showed significant improvement in children’s reading skills. Parents were trained and supervised to use specific techniques to improve their children’s reading skills. The programme aimed not only to improve the parents’ technical skills, but also to create a more effective educational environment for the children. The results of the study showed a significant improvement in the children’s reading skills. This suggests that parents, with appropriate training and supervision, can play a crucial role in the treatment of reading difficulties, even in the most severe cases. However, the lack of a control group and the variable starting conditions of the participants limit the generalizability of the results. The study by Ghaffari et al. (2022) introduced an approach combining OPCwith the 4QM. Results suggest that the programme not only improves participation in extracurricular activities, but also improves parents’ ability to meet their children’s learning needs. Ruan et al. (2022) implemented an online course for parents to improve literacy skills. The one-month Massive Open Online Course (MOOC) programme showed significant improvements in children’s skills, highlighting the key role of parents in education. Limitations of the study include the parents’ self-assessment of the completion of the course and the lack of direct monitoring of the quality of teaching, in contrast to Ryback’s study above, which involved close supervision. The study by Shechtman and Gilat (2005) analysed a psycho-educational intervention that provided information and techniques to parents of children with learning difficulties. Although the programme did not produce significant improvements in parental stress or relationships with the child, it highlighted the need to include additional emotional components to achieve more significant results. Finally, Uslu et al. (2006) conducted a one-month in-person psycho-educational intervention that provided parents with information about learning difficulties and their behavioural impact. The results showed an improvement in the children’s reading skills. In summary, the reviewed studies emphasise the transformative role that parents can play in their children’s education when equipped with appropriate skills, emotional support, and guidance. Interventions that balance practical training with relational and emotional components are more likely to yield significant, with lasting improvements in children’s competencies. These findings highlight the importance of designing holistic programmes that empower parents as both educators and emotionally attuned caregivers, ensuring a cascading positive effect on children’s developmental and educational outcomes.

#### 3.3.6 Emotional and relational interventions to support parents

Interventions with an emotional approach aim to reduce stress, increase emotional awareness and improve the overall well-being of parents. Brock and Shute (2013) developed an online programme combining coping strategies and behaviour management for parents. In four weekly two-hour sessions, mothers reported a reduction in parenting stress. However, almost half maintained clinical levels of stress, suggesting that although the programme is useful, further research is needed. George et al. (2011) adapted the Incredible Years programme for parents of children with learning disabilities. The 12 sessions of 2 hours each reduced parental stress and promoted more positive behaviour in the children, but a small increase in behavioural problems was also observed. Multhauf et al. (2016) proposed a CBT intervention delivered in five sessions over 3 months. This approach significantly reduced parental stress, improved conflict management and promoted more positive parent–child interactions. The beneficial effects were maintained after 3 months, highlighting the robustness of the intervention. Shechtman and Gilat (2005) integrated a counselling intervention for parents and reported significant improvements in coping and relationships with their children, whereas the psycho-educational intervention showed no significant benefits. The study highlights the importance of emotional support in group coping programmes. Lastly, Tan et al. (2024) developed an online mindful parenting programme based on mindfulness practices and experiential exploration. The eight weekly two-hour sessions reduced parenting stress, improved emotional awareness and promoted self-compassion. In conclusion, the reviewed interventions demonstrate that addressing parents’ emotional well-being and relational dynamics is crucial for fostering effective parenting and supporting children’s development. While many programmes successfully reduce stress, enhance emotional awareness, and improve parent–child interactions, some limitations persist, such as residual clinical stress levels or mixed behavioural outcomes in children. These findings underscore the need for continued refinement of emotional and relational approaches, ensuring they are robust, adaptable, and capable of addressing the diverse needs of families. By prioritising emotional support and fostering self-compassion, these interventions can create a more resilient and nurturing environment, benefiting both parents and their children.

## 4 Discussion

This systematic review aimed to highlight the variety and effectiveness of interventions developed for supporting parents of children with specific learning disabilities (SLD). The qualitative analysis of the included studies suggests that existing interventions seem to have clear benefits for parents in terms of emotional well-being, stress reduction and improved parent–child relationships, and for children in terms of academic and behavioural outcomes. Specifically, the interventions in the literature are online or in-person, individual or group, with an emotional or didactic focus.

### 4.1 Online and in-person interventions

Online interventions, such as those by Abd Rauf et al. (2020) and Tan et al. (2024), demonstrated accessibility and flexibility, benefiting parents who may have limited access to in-person programmes. Furthermore, both offer the opportunity to overcome geographical and time constraints. These interventions have been effective in reducing parental stress and providing strategies for managing children’s difficulties at school. However, they often lack direct supervision, which may affect consistency of implementation. In contrast, in-person interventions, as highlighted by Ghaffari et al. (2022) and George et al. (2011), provide a structured environment that encourages direct support and interaction. Both programmes (online and in-person) have been shown to be particularly effective in improving outcomes for parents and children, although they require more resources and commitment from parents.

### 4.2 Individual vs. group interventions

Group interventions promoted shared experiences and peer support, fostering a sense of community among parents. Shechtman and Gilat (2005) highlighted the emotional benefits of group counselling, which improved coping and parent–child relationships. On the other hand, individual interventions, such as Ryback and Staats (1970) SMART technique, provided tailored solutions to specific challenges, particularly in improving literacy, but were resource intensive and less generalizable.

### 4.3 Teaching and emotional focus

Interventions that combined practical teaching strategies with emotional support produced the most significant results. For example, the Occupational Performance Coaching model used by Ghaffari et al. (2022) not only improved children’s participation in extracurricular activities, but also enhanced mothers’ skills in addressing educational needs. Similarly, programmes such as CBT-based intervention (Multhauf et al., 2016) and Mindful Parenting (Tan et al., 2024) demonstrated sustained reductions in stress and improvements in emotional awareness and parent–child interactions.

## 5 Limitations

Despite the success of many interventions, some studies have reported persistent clinical levels of stress among parents (e.g., Brock and Shute, 2013). This suggests that current programmes may need to include more comprehensive or extensive emotional support components. One of the variables that could be worked on is co-parenting. Indeed, good co-parenting is associated with less stress for the child and a more stable environment. This is due to the sharing of responsibilities, which can reduce the burden on each parent. Furthermore, the vicious circle of child behavioural problems, parental stress and negative parental behaviour is a self-perpetuating mechanism that creates a cycle that is difficult to break. The repetitive and reinforcing cycle leads to feelings of being unable to control or help the child and feelings of guilt for the child’s behaviour or for not knowing how to deal with it. One solution, not mentioned in the above services, could be to strengthen the parent–child bond through shared activities that build trust, communication and mutual affection. In addition, methodological limitations, including small sample sizes, lack of control groups and variability in participant characteristics, limit the generalizability of the findings.

## 6 Conclusion and implications for future research

The analysis highlights the need for interventions for parents of children with SLD to effectively support both the parent and the child. Evidence shows that such interventions improve parents’ emotional well-being by reducing stress levels and increasing self-compassion. This improvement is often linked to appropriate psychoeducation, which increases caregivers’ understanding of their child’s difficulties and equips them with effective strategies. Tailored interventions that combine emotional and practical support have been shown to be effective in improving the overall quality of family life. Both group and individual approaches offer unique strengths, while online and hybrid models offer opportunities to overcome geographical and time constraints. However, ensuring quality and engagement in these models remains critical. Findings from this review have the potential to inform future programmes that should consider balancing emotional and practical support, increasing accessibility and inclusivity, especially for under-represented populations, including mechanisms for sustained follow-up to ensure long-term benefits, expanding research on hybrid intervention models and their effects over time. While interventions significantly improve parental well-being and coping strategies, some parents continue to experience stress, suggesting the need for further refinement. Effective support systems not only promote emotional resilience in parents, but also create a stable and nurturing environment for children, contributing to their academic success and overall development. This dual focus on parents and children is essential for fostering long-term positive outcomes for families. It is important to emphasise that any intervention designed to support parents of children with SLD is valuable as long as it contributes to their well-being. Whether through psychoeducation, emotional support, practical strategies or a combination of these, the focus should remain on the positive impact such interventions have on the parents themselves. By prioritising parents’ emotional resilience and self-compassion, these programmes empower them to better cope with the challenges they face, ultimately fostering healthier and more supportive family dynamics. Parental well-being is not only a worthy goal, but also a critical foundation for creating an environment in which children can thrive academically, socially and emotionally.

## Data Availability

The original contributions presented in the study are included in the article/Supplementary material, further inquiries can be directed to the corresponding authors.
