# Supplementary material for: Supporting parents of children with learning disorders: a systematic review of intervention strategies
Source: Front Psychol. 2025 May 30;16:1536894. doi: 10.3389/fpsyg.2025.1536894 (PMC12163006; doi:10.3389/fpsyg.2025.1536894)

Supplementary Material

S.1. Search strategy

The results of the identification process in the search string format can be referenced in Table 1.

Table 1| Results of the identification process in search string format.

Databases	Search string	Number
**Web of Science**	TS= ("specific learning disorder" OR "specific learning impairment" OR dyslex* OR dyscalcul* OR dysgraph* OR dysorthograph*) AND TS=(parent* OR mother* OR father* OR famil* OR caregiver*)AND TS=(support* OR intervention* OR training* OR counsel* OR empower* OR "parental involvement" OR "family support")NOT TS=(autism OR ADHD OR "intellectual disability")	671
**Scopus**	(TITLE-ABS("specific learning disorder" OR "specific learning impairment" OR dyslex* OR dyscalcul* OR dysgraph* OR dysorthograph*)) AND (TITLE-ABS(parent* OR mother* OR father* OR famil* OR caregiver*)) AND (TITLE-ABS(support* OR intervention* OR training* OR counsel* OR empower* OR "parental involvement" OR "family support")) AND NOT (TITLE-ABS(autism OR ADHD OR "intellectual disability"))	564
**PubMed**	((specific learning disorder[Title/Abstract] OR specific learning impairment[Title/Abstract] OR dyslex*[Title/Abstract] OR dyscalcul*[Title/Abstract] OR dysgraph*[Title/Abstract] OR dysorthograph*[Title/Abstract])) AND (parent*[Title/Abstract] OR mother*[Title/Abstract] OR father*[Title/Abstract] OR famil*[Title/Abstract] OR caregiver*[Title/Abstract]) AND (support*[Title/Abstract] OR intervention*[Title/Abstract] OR training*[Title/Abstract] OR counsel*[Title/Abstract] OR empower*[Title/Abstract] OR "parental involvement"[Title/Abstract] OR "family support"[Title/Abstract]) NOT (autism[Title/Abstract] OR ADHD[Title/Abstract] OR "intellectual disability"[Title/Abstract])	284

S.2. Study risk of bias assessment

The Cochrane Risk of Bias Tools – 2 were used to assess bias. The authors preferred the RoB 2 (updated version), which is more rigorous to assist the reviewer in making a judgement. The two authors worked independently and allocated the selected articles equally and randomly. Furthermore, to further verify the degree of agreement between the evaluators, Cohen's index k was calculated, which resulted as equal to 1, indicating a perfect agreement between the evaluators. A summary ROB score (low, unclear, high) was assigned to each study, based on the following criteria: appropriateness of randomisation; allocation of treatment deviations; handling of dropout data and incomplete outcome data; outcome measurement; reported outcome. The summary ROB scores are defined as follows [*Fig 1*]:

- ROB low: bias, if present, is unlikely to seriously alter the results.

- ROB unclear: risk of bias that casts doubt on the results.

- ROB high: bias may seriously affect the results.

Figure. 1| Risk of Bias Assessment Across Included Studies

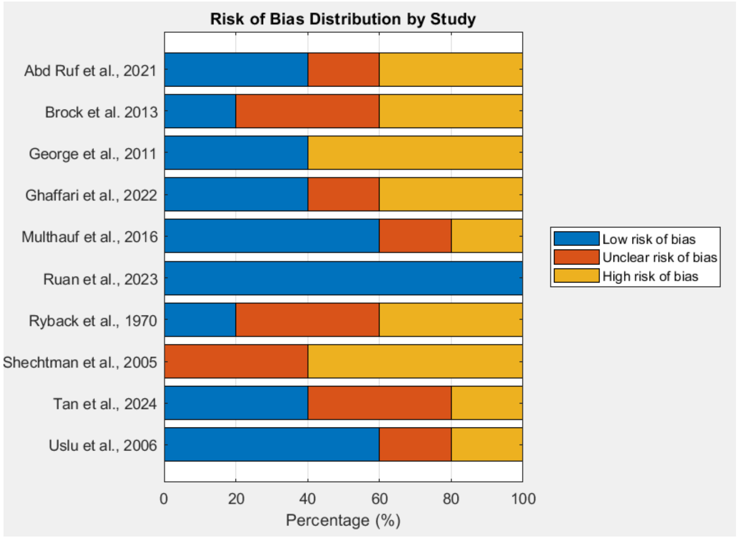

Supplement: Supplementary file 1 [file Data_Sheet_1.PDF]
